# Environment-Driven Synthetic Baseline Analysis and Optimization in Joint Measurement OPM-MEG Arrays

**DOI:** 10.3390/bioengineering13060599

**Published:** 2026-05-22

**Authors:** Wenli Wang, Jianxin Yang, Weinan Xu, Fuzhi Cao, Nan An, Zhenfeng Gao, Min Xiang, Wen Li

**Affiliations:** 1 Key Laboratory of Ultra-Weak Magnetic Field Measurement Technology, Ministry of Education, School of Instrumentation and Optoelectronic Engineering, Beihang University, Beijing 100191, China; zb1917006@buaa.edu.cn (W.W.); sy2354126@buaa.edu.cn (J.Y.); xwnan@buaa.edu.cn (W.X.); caofuzhi@buaa.edu.cn (F.C.); gaozhenfeng@buaa.edu.cn (Z.G.); 2 Hangzhou Institute of National Extremely-Weak Magnetic Field Infrastructure, 465 Binan Rd., Binjiang District, Hangzhou 310051, China; annan@buaa.edu.cn; 3 Hefei National Laboratory, 96 Jinzhai Rd., Gaoxin District, Hefei 230088, China

**Keywords:** magnetoencephalography, OPM-MEG, joint measurement sensor array, synthetic baseline, source localization

## Abstract

Optically pumped magnetometer-based magnetoencephalography (OPM-MEG), with its flexible sensor configuration and wide range of application scenarios, has become a powerful complement to conventional superconducting quantum interference device magnetoencephalography (SQUID-MEG). However, this higher flexibility also means that OPM-MEG sensor arrays are more susceptible to interference from complex and variable background magnetic noise. Previous research has shown that deploying reference sensors around the scalp array for noise cancellation is an effective strategy. Nonetheless, the selection of its key parameter, the spatial distance between the reference and scalp sensors, commonly termed the synthetic baseline, predominantly relies on empirical rules and lacks systematic theoretical optimization. To address this issue, this study thoroughly investigates the fundamental impact of the synthetic baseline on the system’s noise suppression performance. Simulation results demonstrate that the optimal baseline length is not a fixed value but varies systematically with environmental noise characteristics and the specific requirements of the source localization task. Building on this analysis, a Baseline Adaptive Reference Optimization (BARO) method is proposed. As an environment-driven strategy, the BARO method automatically determines the optimal baseline configuration by maximizing the output signal-to-noise ratio (SNR). Compared to traditional fixed-baseline configurations, the proposed BARO method significantly enhances the output SNR and effectively reduces the localization error of equivalent current dipoles within the brain across various simulated complex noise scenarios. This work provides a physically interpretable criterion for baseline optimization and offers theoretical support for environment-adaptive configuration of OPM-MEG sensor arrays.

## 1. Introduction

Magnetoencephalography (MEG) is a noninvasive neuroimaging technique that records the extremely weak magnetic fields generated by brain activity and offers high temporal and spatial resolution [1,2]. The rapid development of optically pumped magnetometers (OPMs) is driving MEG technology to transition from traditional measurement systems that rely on cryogenic superconducting quantum interference devices (SQUIDs) toward room-temperature, wearable, and flexibly configurable modalities [3,4,5]. Compared with SQUID-based MEG systems, OPM sensors do not require cryogenic cooling and can be placed closer to the scalp, thereby significantly increasing signal amplitude and improving adaptability for measurements in children, individuals with atypical head shapes, and during naturalistic behaviors [6,7,8]. However, the increased proximity to the scalp, while enhancing signal strength, also places higher demands on the precision of interference suppression, as localized gradients and motion-induced artifacts become more prominent relative to the neural signal [9,10]. Therefore, achieving effective suppression of environmental interference while maintaining sensitivity to neural signals has become a central challenge in the design of OPM-MEG sensor arrays.

Currently, the mainstream noise suppression strategy in OPM-MEG employs a joint measurement configuration consisting of a scalp array and a small number of reference sensors [11,12]. This strategy can significantly reduce common-mode interference and improve the signal-to-noise ratio (SNR) in non-ideal environments [13,14,15]. The core mechanism relies on the high spatial coherence of far-field environmental noise across both arrays. While the reference sensors characterize these shared noise components, the localized near-field neural signals remain predominantly present in the scalp array due to their rapid spatial decay. This spatial differentiation allows for the effective subtraction of common-mode interference through regression or signal space separation algorithms while preserving neural signal fidelity [16,17]. However, in contrast to the rapid development at the algorithmic level, the spatial layout design of reference sensor arrays remains largely empirical, and in particular, a unified theoretical analysis framework for the spatial distance between the reference sensors and the primary array is still lacking. This spatial distance, namely the synthetic baseline distance, essentially determines the capability of the system to sample the spatial gradient of the environmental magnetic field and serves as a key physical parameter influencing the trade-off between noise suppression efficiency and neural signal fidelity [18]. When the baseline distance is too small, it is difficult for environmental interference to form an effective difference; when the baseline distance is too large, the spatial coherence of the environmental interference between the scalp and reference arrays may decrease, leading to reduced noise cancellation efficiency and potential instability in the signal reconstruction [19,20]. Therefore, it is necessary to systematically investigate the influence of the synthetic baseline on measurement performance and to establish a generally applicable optimization design method.

To reveal the fundamental influence of the spatial distance of reference sensors on system performance, this study conducts a systematic investigation centered on the synthetic baseline. First, the variation patterns in performance under different noise statistical characteristics are analyzed. Based on this analysis, a Baseline-Adaptive Reference Optimization (BARO) approach is introduced as an environment-driven baseline selection strategy. This approach identifies an optimal synthetic baseline by maximizing the output SNR under given conditions. Simulation results demonstrate that the optimal synthetic baseline is not a constant but varies systematically with environmental noise characteristics. Compared to fixed empirical configurations, this strategy improves array SNR and reduces source localization errors across different simulated scenarios.

## 2. Materials and Methods

### 2.1. Measurement Model

In a configuration where the scalp array and the reference array jointly participate in the measurement, it is assumed that the measurement data consist of one intracranial target source, one extracranial interference source, environmental noise, and sensor floor noise. Under this assumption, the joint measurement equation at time *t* is expressed as follows:

(1) $$m(t;d)=q_{\mathrm{int}}L_{\mathrm{int}}(d)s_{\mathrm{int}}(t)+m_{\mathrm{ext}}(t;d)+m_{\mathrm{env}}(t;d)+m_{\mathrm{base}}(t)$$

Here, $m(t;d)\in {\mathbb{R}}^{M\times 1}$ is the measurement vector, where *M* is the total number of sensors. $L_{\mathrm{int}}(d)=[L_{\mathrm{int}}^{\mathrm{scalp}};L_{\mathrm{int}}^{\mathrm{ref}}(d)]\in {\mathbb{R}}^{M\times 3}$ represents the joint lead field matrix of the intracranial target source, where $L_{\mathrm{int}}^{\mathrm{scalp}}\in {\mathbb{R}}^{N_{\mathrm{scalp}}\times 3}$ and $L_{\mathrm{int}}^{\mathrm{ref}}(d)\in {\mathbb{R}}^{N_{\mathrm{ref}}\times 3}$ denote the lead field components for the scalp array and the reference array, respectively ($M=N_{\mathrm{scalp}}+N_{\mathrm{ref}}$). Terms $m_{\mathrm{ext}}(t;d)\in {\mathbb{R}}^{M\times 1}$ and $m_{\mathrm{ext}}(t;d)\in {\mathbb{R}}^{M\times 1}$ respectively represent the noise from external interference sources and the environmental noise. Specifically, $m_{\mathrm{ext}}(t;d)$ refers to localized extracranial interference from sources near the subject, such as the magnetic fields generated by the heart (magnetocardiography, MCG) or muscular artifacts. $m_{\mathrm{env}}(t;d)$ represents far-field environmental noise originating from distant sources, such as power line interference (50/60 Hz), nearby moving vehicles, or elevators, which typically exhibits high spatial coherence across the sensor array. The Term $m_{\mathrm{base}}(t)\in {\mathbb{R}}^{M\times 1}$ represents the sensor floor noise, which is typically approximated as white noise.

Based on the aforementioned model, the overall covariance is decomposed into the sum of the intracranial target source term and the total noise term:

(2) $$C(d)=q_{\mathrm{int}}^{2}L_{\mathrm{int}}(d)L_{\mathrm{int}}^{T}(d)+\Sigma (d)$$

In this expression, Σ(d) is the generalized noise covariance, which includes the extracranial interference source, environmental noise, and floor noise. To emphasize the structure between the scalp array and the reference array, the block matrix form is denoted as:

(3) $$\Sigma (d)=\left[\begin{array}{cc} \Sigma_{\mathrm{MM}}(d) & \Sigma_{\mathrm{MR}}(d)\\ \Sigma_{\mathrm{RM}}(d) & \Sigma_{\mathrm{RR}}(d) \end{array}\right]$$

where the cross-term $\Sigma_{\mathrm{MR}}(d)$ describes the noise correlation between the scalp array and the reference array, which is the critical factor for noise suppression facilitated by the reference array.

Given the lead field $L_{\mathrm{int}}(d)$ of the intracranial target source, the Linearly Constrained Minimum Variance (LCMV) constraint is defined as [21]:

(4) $$w^{T}(d)L_{\mathrm{int}}(d)=1$$

By utilizing the generalized noise covariance Σ(d) to construct the optimal noise suppressor, the LCMV optimization problem is formulated as:

(5) $$\underset{w}{\min}\;w^{T}\Sigma (d)w\;s.t.\;w^{T}L_{\mathrm{int}}(d)=1$$

The closed-form solution is given by:

(6) $$w(d)=\frac{\Sigma^{-1}(d)L_{\mathrm{int}}(d)}{L_{\mathrm{int}}^{T}(d)\Sigma^{-1}(d)L_{\mathrm{int}}(d)}$$

The output of the beamformer is:

(7) $$\hat{q}(t;d)=w^{T}(d)m(t;d)$$

According to the constraint $w^{T}L_{\mathrm{int}}(d)=1$, the output signal power is determined to be:

(8) $${\mathrm{var}}_{\mathrm{signal}}=q_{\mathrm{int}}^{2}$$

The output noise power is expressed as:

(9) $${\mathrm{var}}_{\mathrm{noise}}(d)=w^{T}(d)\Sigma (d)w(d)=\frac{1}{L_{\mathrm{int}}^{T}(d)\Sigma^{-1}(d)L_{\mathrm{int}}(d)}$$

Consequently, the output SNR is calculated as:

(10) $$\mathrm{SNR}(d)=q_{\mathrm{int}}^{2}L_{\mathrm{int}}^{T}(d)\Sigma^{-1}(d)L_{\mathrm{int}}(d)$$

By substituting the decomposition form of the lead field, the following equation is obtained:

(11) $$\mathrm{SNR}(d)=q_{\mathrm{int}}^{2}{\left[\begin{array}{c} L_{\mathrm{int}}^{\mathrm{scalp}}\\ L_{\mathrm{int}}^{\mathrm{ref}}(d) \end{array}\right]}^{T}\Sigma^{-1}(d)\left[\begin{array}{c} L_{\mathrm{int}}^{\mathrm{scalp}}\\ L_{\mathrm{int}}^{\mathrm{ref}}(d) \end{array}\right]$$

Since the position of the scalp array remained fixed, $L_{\mathrm{int}}^{\mathrm{scalp}}$ served as a constant. The positional change of the reference array influenced the beamforming output through the joint effect of $L_{\mathrm{int}}^{\mathrm{ref}}(d)$ and the noise covariance Σ(d), thereby determining the overall imaging performance. Therefore, by adjusting the distance *d* between the reference array and the scalp array to maximize the output SNR, the output noise power of the beamformer was reduced, which subsequently improved the source localization accuracy of the LCMV.

### 2.2. Noise Model

To accurately quantify the various interference terms in the measurement model, mathematical modeling was performed for the extracranial interference sources, the environmental noise and the sensor floor noise.

The noise from external interference sources $m_{\mathrm{ext}}(t;d)$ was modeled as external point source interference with intensity $q_{\mathrm{ext}}$. At time *t*, the measurement contribution generated by this interference at the sensor array was expressed as follows:

(12) $$m_{\mathrm{ext}}(t;d)=q_{\mathrm{ext}}L_{\mathrm{ext}}(d)s_{\mathrm{ext}}(t)$$

where $L_{\mathrm{ext}}(d)$ represented the lead field of the extracranial interference source on the joint array, the value of which depended on the source position and the geometric topology of the array.

**Figure 1:**
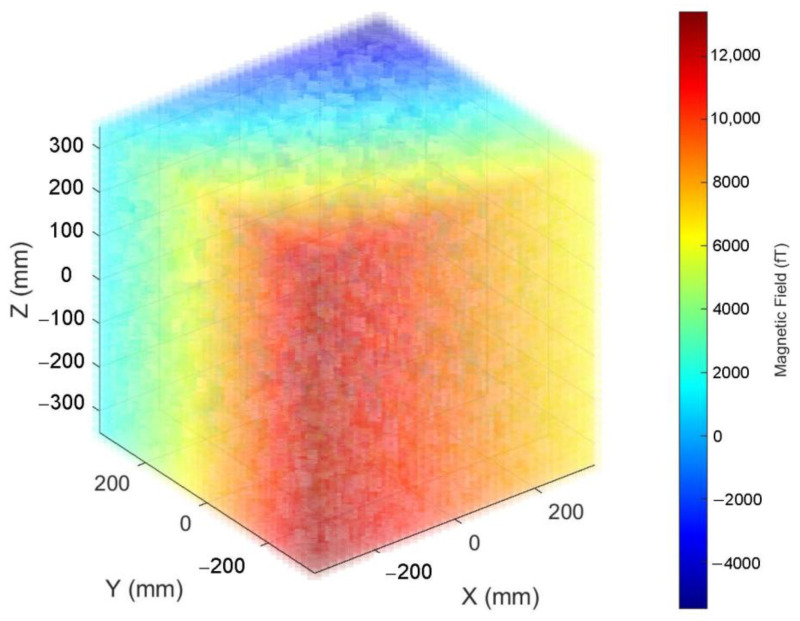
Schematic diagram of environmental noise.

The characterization of the environmental noise power spectral density was based on the theoretical framework proposed by Vrba [22] (Figure 1). This model assumed that the environmental noise $m_{\mathrm{env}}(f)$ exhibited a characteristic $1/f^{k}$ spectrum at low frequencies ($f<f_{\mathrm{ob}}$), which was expressed by the following mathematical equation:

(13) $$m_{\mathrm{env}}(f)=\frac{A}{f^{k}}\;$$

where *A* represented a constant for noise intensity, and *k* denoted the slope of the noise power in a log-log representation. Conversely, the sensor floor noise associated with the OPM sensors was modeled in the frequency domain as a frequency-independent white noise density, denoted as $m_{\mathrm{base}}$ with the unit of $\mathrm{fT}\cdot {\mathrm{Hz}}^{-1/2}$. The corner frequency $f_{\mathrm{ob}}$ was defined as the intersection where the environmental noise intensity equaled the sensor floor noise intensity, which was derived as follows:

(14) $$m_{\mathrm{base}}=m_{\mathrm{env}}(f_{\mathrm{ob}})\Rightarrow m_{\mathrm{base}}=\frac{A}{f_{\mathrm{ob}}^{k}}\;$$

In the design of the magnetic gradiometer, a core physical assumption was that the environmental noise amplitude captured by the gradiometer was directly proportional to the baseline length *d*. Given a reference baseline $d_{0}$ and the corresponding noise constant $A_{0}$, the environmental noise constant *A* for any arbitrary baseline *d* was defined as:

(15) $$A=A_{0}\frac{d}{d_{0}}\;$$

Based on this proportional relationship, the corner frequency $f_{\mathrm{ob}}$ also underwent a nonlinear shift with the baseline length according to the following law:

(16) $$f_{\mathrm{ob}}=f_{0}{\left(\frac{d}{d_{0}}\right)}^{k/2}\;$$

In this equation, $f_{0}$ represented the initial corner frequency measured at the reference baseline $d_{0}$, the value of which reflected the magnetic shielding effectiveness of the measurement environment. Equation (16) indicated that as the baseline *d* increased, the dominance region of the 1/f noise shifted toward higher frequencies, resulting in a broader frequency band of interest being obscured by environmental interference. These spectral density functions, $m_{\mathrm{env}}(f)$ and $m_{\mathrm{base}}$, characterized the statistical properties of the time-domain noise terms $m_{\mathrm{env}}(t;d)$ and $m_{\mathrm{base}}(t)$ presented in Equation (1).

### 2.3. Reference Sensor Model

**Figure 2:**
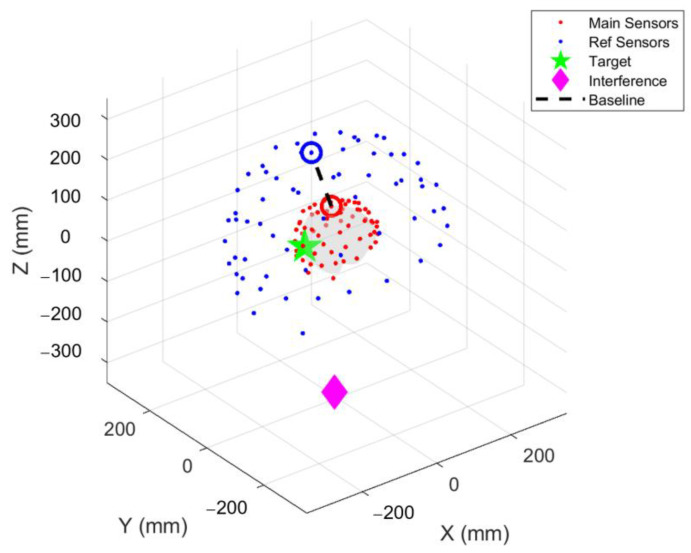
Schematic diagram of reference sensors.

To analyze the physical mechanisms between the baseline length *d* and various noise sources intuitively, the reference array model was simplified through engineering approximations into a standard first-order radial gradiometer model (Figure 2). Under this model, the beamforming weights were fixed in a physical differential form. It was assumed that the sensor floor noise, environmental noise, and external point source interference were statistically independent. Consequently, the quadratic operation of the generalized noise covariance matrix was simplified into a scalar superposition of the variance of each noise component. Based on these simplifications, the general SNR in Equation (10) was expressed in the following form:

(17) $$\mathrm{SNR}(d;f_{0},q_{\mathrm{ext}})\approx \frac{S_{\mathrm{int}}^{2}(d)}{N_{\mathrm{base}}^{2}+N_{\mathrm{env}}^{2}(d;f_{0})+N_{\mathrm{ext}}^{2}(d;q_{\mathrm{ext}})}\;$$

where $S_{\mathrm{int}}^{2}(d)$ represented the effective signal amplitude of the intracranial target source under the first-order radial gradiometer mode. $N_{\mathrm{base}}^{2}$ denoted the fixed sensor floor noise power, which was derived by integrating the OPM sensor floor noise density $m_{\mathrm{base}}$ over the analysis bandwidth. $N_{\mathrm{env}}^{2}(d;f_{0})$ represented the environmental noise power, the intensity of which was determined by the shielding level parameter $f_{0}$ (the initial corner frequency) and the baseline dependency defined in Equation (16). $N_{\mathrm{ext}}^{2}(d;q_{\mathrm{ext}})$ characterized the residual amount of the external point source interference with intensity $q_{\mathrm{ext}}$ after the differential process.

By adjusting the shielding level $f_{0}$ and the interference intensity $q_{\mathrm{ext}}$, the nonlinear effects of environmental noise and external interference sources on the optimal baseline were quantified clearly. Specifically, when the parameters $f_{0}$ and $q_{\mathrm{ext}}$ were both zero, the entire measurement system was in an ideal shielded state without external interference. As $f_{0}$ and $q_{\mathrm{ext}}$ increased, the shielding performance declined and the external interference signals strengthened. This model revealed the manner in which the optimal baseline sought a dynamic equilibrium between signal capture capability and interference suppression capability to adapt to different clinical environments.

### 2.4. Baseline Optimization

The Baseline Optimization for Reference Orientation (BARO) method was employed to determine the optimal baseline distance $d_{\mathrm{opt}}$ between the scalp array and the reference array to adapt to diverse imaging tasks and noise conditions. Specifically, the OPM gradiometer array was assumed to operate under specific environmental noise conditions, which were jointly parameterized by the environmental noise corner frequency $f_{0}$ and the external interference source intensity $q_{\mathrm{ext}}$. For the entire sensor array or an individual gradiometer unit, the baseline length *d* was allowed to be adjusted within a certain range. The initial length of the baseline was determined by a universal configuration, such as *d* = 5 cm. To achieve baseline optimization for the current imaging task, the baseline length was traversed within a predefined discrete search space.

The candidate set for baseline lengths was defined as $D=\{d^{\left(1\right)},d^{\left(2\right)},\ldots ,d^{\left(M\right)}\}$, for example, ranging from 1 to 20 cm with a step size of 1 cm. For each candidate baseline length $d^{\left(c\right)}$, where c∈{1,2,…,M}, the corresponding comprehensive performance index $J(d^{\left(c\right)}|\Phi )$ was calculated based on the currently detected environmental parameters $\Phi =\{f_{0},q_{\mathrm{ext}}\}$ and the preset brain source model $q_{\mathrm{int}}$. This index was defined as the estimated beamforming signal to noise ratio under the current configuration, and its calculation form was as follows:

(18) $$J(d^{\left(c\right)})=\frac{S_{\mathrm{int}}^{2}(d^{\left(c\right)})}{N_{\mathrm{total}}^{2}(d^{\left(c\right)})}=\frac{\|L_{\mathrm{int}}(d^{\left(c\right)})\cdot q_{\mathrm{int}}{\|}^{2}}{N_{\mathrm{base}}^{2}+N_{\mathrm{env}}^{2}(d^{\left(c\right)};f_{0})+N_{\mathrm{ext}}^{2}(d^{\left(c\right)};q_{\mathrm{ext}})}\;$$

where the numerator term $\|L_{\mathrm{int}}(d^{\left(c\right)})\cdot q_{\mathrm{int}}{\|}^{2}$ represented the concrete computational implementation of the effective signal amplitude $S_{\mathrm{int}}^{2}$ as defined in Equation (17) within the beamformer. The denominator term was composed of the superposition of the sensor floor noise $N_{\mathrm{base}}^{2}$, the environmental gradient noise $N_{\mathrm{env}}^{2}$ that increased nonlinearly with the baseline, and the residual of the external near field interference $N_{\mathrm{ext}}^{2}$.

The optimal baseline length was subsequently selected as the configuration that maximized this global index:

(19) $$d_{\mathrm{opt}}={\mathrm{argmax}}_{d^{\left(c\right)}\in D}J(d^{\left(c\right)})\;$$

The optimal baseline $d_{\mathrm{opt}}$ served as the final configuration parameter to guide the hardware baseline adjustment or the weight redistribution for software synthetic gradients. This approach ensured that the system achieved optimal global performance within the current specific noise competition scenario.

### 2.5. Simulation Experiment

To systematically evaluate the physical mechanism, effectiveness, and application value in source localization tasks of the BARO method based on optimal suppression of noise interference, three groups of simulation experiments were designed in this section. All experiments relied on a unified OPM-MEG joint measurement framework. Under the premise of strictly maintaining consistency in array geometry, source configuration, and signal processing procedures, only the noise parameters were adjusted specifically to ensure the comparability and consistency of the experimental conclusions.

#### 2.5.1. Sensor Array

To investigate the influence of baseline length variations on the performance of the OPM-MEG joint measurement array, multiple sets of sensor arrays with different baseline length configurations were generated. Specifically, the sensor positions of the scalp array were determined based on the electrode layout of a 64-channel EEG system from BioSemi B.V., Amsterdam, Netherlands, with the sensitive axis directions uniformly oriented radially outward along the head surface. Subsequently, while maintaining the scalp array positions as fixed, the reference array was constructed by translating each sensor outward along its respective radial axis. The translation distance increased from 1 cm to 20 cm in steps of 1 cm, resulting in the generation of 20 different sets of reference array configurations.

#### 2.5.2. Head and Source Models

To construct the head model and source model for lead field calculation, T1-weighted MRI data were collected from a 26-year-old healthy female subject using a 3T magnetic resonance scanner from Prisma, Siemens, Erlangen, Germany. The FreeSurfer software (Version 6) [23] was utilized to segment the MRI data. The Brainstorm software [24] was utilized to extract the brain surface and the cortical surface. The segmented brain surface was employed to construct the head model, while the cortical surface was partitioned into a triangular mesh containing 15,002 vertices to serve as the source model. Each vertex in the mesh represented a potential location for target source activity, and the orientation of the source was constrained to be perpendicular to the local cortical surface [25].

#### 2.5.3. Evaluation Metrics

To systematically evaluate the overall performance of the optimized OPM joint measurement array constructed via the BARO method, three representative evaluation metrics were selected from both the sensor level and the source level: the array SNR (${\mathrm{SNR}}_{\mathrm{array},\mathrm{linear}}$), the relative performance gain (RPG), and the dipole localization error (DLE).

The ${\mathrm{SNR}}_{\mathrm{array},\mathrm{linear}}$ was defined as the ratio of the average power of the output signal to the noise power of the joint measurement array. The differential measurement data at time *t* for *N* joint sensor channels, denoted as $m_{\mathrm{diff}}(t)$, was defined as the linear superposition of the clean differential signal component $m_{\mathrm{signal}}(t)$ and the pure noise component $m_{\mathrm{noise}}(t)$. The calculation formula for the array SNR was as follows:

(20) $${\mathrm{SNR}}_{\mathrm{array},\mathrm{linear}}=\frac{P_{\mathrm{signal}}}{P_{\mathrm{noise}}}=\frac{\sum_{t\in T_{\mathrm{act}}}\|m_{\mathrm{signal}}(t){\|}_{F}^{2}}{\sum_{t\in T_{\mathrm{base}}}\|m_{\mathrm{diff}}(t){\|}_{F}^{2}}\times \frac{N_{\mathrm{base}}}{N_{\mathrm{act}}}\;$$

where $\|\cdot {\|}_{F}$ represented the Frobenius norm, which denoted the sum of energy across all channels. $T_{\mathrm{act}}$ and $T_{\mathrm{base}}$ denoted the signal activation period and the baseline period, respectively. The term $N_{\mathrm{base}}/N_{\mathrm{act}}$ served as a time-window normalization factor to eliminate differences in period lengths, thereby ensuring that the metric characterized the average power ratio rather than the accumulated energy ratio.

The logarithmic form of this metric, measured in decibels, was defined as follows:

(21) $${\mathrm{SNR}}_{\mathrm{array},\mathrm{dB}}=10\log_{10}({\mathrm{SNR}}_{\mathrm{array},\mathrm{linear}})\;$$

The RPG was intended to provide a direct comparison of the performance improvement achieved by the BARO method relative to traditional empirical designs. This metric was calculated based on the aforementioned array SNR as follows:

(22) $$\mathrm{RPG}=\frac{{\mathrm{SNR}}_{\mathrm{array},\mathrm{baseline}}(d_{\mathrm{opt}})-{\mathrm{SNR}}_{\mathrm{array},\mathrm{baseline}}(d_{\mathrm{fix}})}{{\mathrm{SNR}}_{\mathrm{array},\mathrm{baseline}}(d_{\mathrm{fix}})}\times 100\%\;$$

where $d_{\mathrm{fix}}$ represented a designated baseline configuration. A higher RPG value indicated a more significant optimization effect of the BARO method on the baseline configuration under the current noise environment.

The DLE was employed to measure the geometric deviation between the peak position of the source imaging results and the true source position. For a single dipole source located at $r_{\mathrm{true}}$, if the reconstructed source distribution $\hat{q}$ reached its maximum energy or intensity at position $r_{\mathrm{peak}}$, the DLE was defined as the Euclidean distance between the peak coordinate and the true coordinate:

(23) $${\mathrm{DLE}}_{i}=\|r_{\mathrm{peak}}-r_{\mathrm{true}}{\|}_{2}\;$$

where $\hat{q}(r)$ represented the estimated source intensity at position r in the reconstructed source space. A lower DLE value signified that the array could accurately lock onto the anatomical location of neural activity under noise interference.

#### 2.5.4. Simulation Scenarios

(1) Physical mechanisms of noise dominant optimization

To analyze the action mechanism of the baseline distance on different noise statistical characteristics within the joint array, a simulation experiment was first designed to perform a comparative analysis between two typical noise scenarios: environmental noise and extracranial interference source noise. Specifically, the task requirement was fixed as a universal full-band MEG measurement task with a starting frequency $f_{1}=0.1$ Hz and a bandwidth Δf=100 Hz. The interference source was fixed at a position 40 cm below the head to simulate magnetocardiography source signals. Scenario A was configured as an environmental noise only case, where the external interference source intensity parameter $q_{\mathrm{ext}}$ was fixed at 0, and the environmental noise characteristic parameter $f_{0}$ was varied across $\left\{0,1,3,5,8,10\right\}$ Hz. Scenario B was configured as an external interference source only case, where the environmental noise parameter $f_{0}$ was fixed at 0, and the external interference source intensity $q_{\mathrm{ext}}$ was gradually increased across {0,1000,3000,5000,8000,10,000} nAm. On this basis, the nonlinear laws of the output SNR as a function of baseline length, varied from 1 to 20 cm with a step size of 1 cm, were calculated and quantitatively analyzed under different noise dominance mechanisms.

(2) Multi-scenario validation of optimization robustness

To verify the optimization effectiveness of the BARO algorithm in complex noise scenarios, test experiments comprising three groups with a total of nine representative cases were designed based on different neuroscience task requirements, physical environment characteristics, and spatial distributions of interference sources.

Group 1: Task Requirement Adaptability. The physical environment parameters were fixed at $f_{0}=5$ Hz, $q_{\mathrm{ext}}=5000$ nAm, and k=1 while the frequency band settings were varied. Scenario C: The starting frequency $f_{1}=0.1$ Hz and the bandwidth Δf=100 Hz were set to simulate a standard universal full-band MEG measurement task. Scenario D: The parameters $f_{1}=0.1$ Hz and Δf=40 Hz were set to simulate evoked response or slow-wave studies significantly affected by environmental 1/f noise. Scenario E: The parameters $f_{1}=30$ Hz and Δf=50 Hz were set to simulate cognitive function or epilepsy high-frequency oscillation studies where environmental noise attenuation was relatively large.

Group 2: Physical Environment Robustness. The task requirements were fixed at $f_{1}=0.1$ Hz and Δf=100 Hz while the environmental noise characteristics were varied. Scenario F: The baseline noise intensities were set to $f_{0}=5$ Hz and $q_{\mathrm{ext}}=5000$ nAm with superior shielding performance represented by k=1.5 to simulate a high-performance magnetically shielded room environment. Scenario G: The baseline noise intensities were set to $f_{0}=5$ Hz and $q_{\mathrm{ext}}=5000$ nAm with inferior shielding performance represented by k=0.8 to simulate an open or lightweight shielded environment. Scenario H: High noise intensities were set to $f_{0}=10$ Hz and $q_{\mathrm{ext}}=10,000$ nAm with inferior shielding performance represented by k=0.8 to simulate high-stress testing under extreme industrial or clinical outpatient environments.

Group 3: Interference Source Position Sensitivity. The environment and task parameters were fixed at $f_{1}=0.1$ Hz, Δf=100 Hz, $f_{0}=5$ Hz, $q_{\mathrm{ext}}=5000$ nAm, and k=1 while the geometric positions of the interference source were varied. Scenario I: The interference source was located 15 cm below the head to simulate near-field interference generated by neck stimulators or similar sources. Scenario J: The interference source was located 40 cm below the head to simulate mid-field baseline interference caused by heart magnetic signals or similar sources. Scenario K: The interference source was located 200 cm away to simulate far-field interference approximating a uniform field.

For each specific scenario mentioned above, the corresponding task frequency bands, environmental constants, and interference source characteristics were input into the BARO algorithm as prior information. A full traversal search was executed within the predefined discrete baseline space, ranging from 1 to 20 cm with a step size of 1 cm, to determine and output the global optimal baseline configuration $d_{\mathrm{opt}}$ for that specific condition.

(3) Source localization accuracy and imaging performance

To evaluate the effectiveness of the optimal baseline in practical source localization, the ‘High-frequency Task’ (Scenario E) was selected from the nine pre-defined interference scenarios to construct a simulated OPM-MEG dataset. This scenario is highly correlated with the energy profiles observed in empirical OPM-MEG recordings. The source signal consisted of a 30 Hz evoked oscillation packet superimposed from multiple Gaussian transient peaks and a 30 to 70 Hz band-pass evoked post-oscillation. The source amplitude was fixed at $q_{\mathrm{int}}=10$ nAm. The external interference source was located 40 cm to the lower left of the brain and was modeled using a standardized MCG time template with an amplitude of $q_{\mathrm{ext}}=5000$ nAm. Environmental noise parameters were set to $f_{0}=5$ Hz and k=1.

In all simulation scenarios, the source model and forward calculation followed a unified physical definition. Target sources were randomly selected from the aforementioned source space model, and a single-shell realistically shaped head model [26] was constructed based on the individual MRI data described in Section 2.5.2. The lead field was calculated using Nolte’s magnetic field calculation method to account for volume conduction effects. External interference sources were modeled as discrete magnetic point dipoles located 15 cm away from the scalp array, and their magnetic fields and lead field matrices were calculated based on the Biot–Savart law in free space [27]. Environmental noise was based on the Vrba empirical model [22], assuming a spatial distribution that was approximately uniform within the array scale and exhibited significant enhancement in the low-frequency band. Inherent sensor noise was modeled as independent Gaussian white noise with a standard deviation of 30 fT.

**Figure 3:**
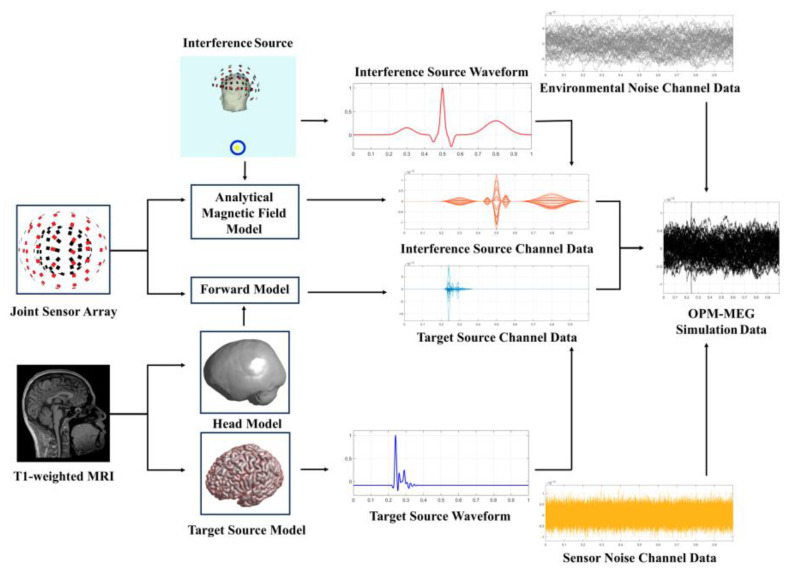
Simulation process of the OPM-MEG data.

The final simulated OPM-MEG data was formed by the superposition of internal signals, external interference signals, environmental gradient noise, and sensor noise, with a total data duration of 1000 ms, where the first 200 ms was utilized as the baseline segment (Figure 3). Source imaging was implemented using the LCMV beamforming method [21] within the FieldTrip toolbox (Version 20220202) [28]. For comparative analysis, multiple baseline configurations were set as references, such as d=1,2,3,…,10 cm, including the optimal baseline obtained through BARO and several commonly used fixed settings. Fifty Monte Carlo random repetitive trials were performed for each simulation condition to summarize the statistical distribution of the DLE and to systematically evaluate the performance advantages of the optimal baseline configuration in improving accuracy.

## 3. Results

### 3.1. Experiment 1: Analysis of the Influence of Different Baseline Lengths on Noise Statistical Characteristics

**Figure 4:**
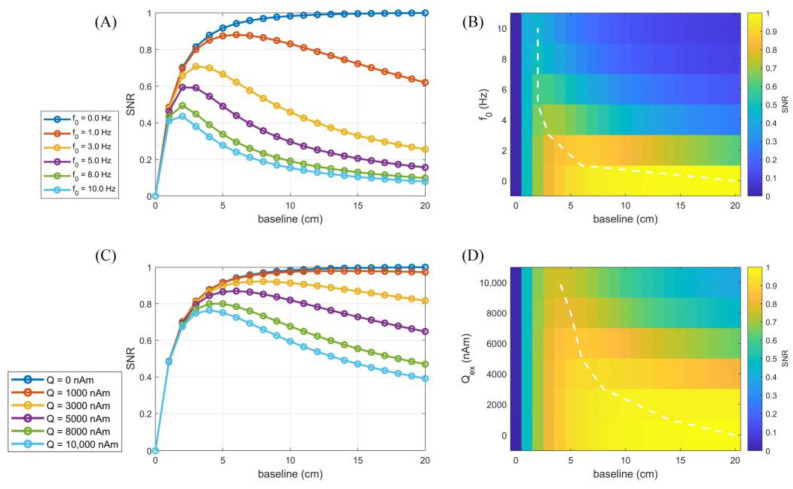
Effects of different baseline lengths on noise statistical characteristics: (**A**) The influence of environmental noise levels on SNR curves. (**B**) SNR heat map in the environmental parameter space. (**C**) The influence of the intensity of external interference sources on the SNR curve. (**D**) SNR heat map in the space of interference intensity.

Figure 4 illustrates the influence of baseline length on the normalized ${\mathrm{SNR}}_{\mathrm{array},\mathrm{linear}}$ of the OPM-MEG joint measurement array and the evolution of the optimal baseline position $d_{\mathrm{opt}}$ under different noise dominance mechanisms. Specifically, Figure 4A shows the relationship between the environmental shielding level, characterized by the environmental noise parameter $f_{0}$, and the system signal to noise ratio. Under ideal shielding conditions where $f_{0}=0$ Hz, represented by the dark blue line, the environmental gradient noise was extremely low. The ${\mathrm{SNR}}_{\mathrm{array},\mathrm{linear}}$ exhibited a monotonic upward trend with increasing baseline length and reached saturation. This indicated that a long baseline where d>20 cm maximized the capture efficiency of brain signals in this scenario. However, as the shielding performance declined, such as when $f_{0}$ increased to 10 Hz as shown by the light blue line, the environmental gradient noise grew significantly with baseline length and rapidly became dominant. Consequently, the curve exhibited a prominent inverted U-shaped characteristic. The optimal baseline position $d_{\mathrm{opt}}$ shifted sharply from a long baseline to the ultra-short baseline region where $d_{\mathrm{opt}}\approx 2$ cm to utilize the strong differential effect for environmental noise suppression. The corresponding heatmap results in Figure 4B further reveal the overall distribution characteristics of the system signal to noise ratio in the two-dimensional parameter space. It was observed that in regions with low $f_{0}$, the SNR maintained a high-value plateau over a wide range of baselines. As $f_{0}$ increased, the high SNR region gradually contracted toward the direction of short baselines, forming a clear optimal baseline trajectory indicated by the white dashed line. This trajectory was consistent with the leftward shift trend of $d_{\mathrm{opt}}$ revealed in the line chart of Figure 4A, demonstrating that the optimal baseline length was highly sensitive to changes in the spatial correlation characteristics of noise under conditions dominated by environmental gradient noise.

Figure 4C further presents the baseline optimization results under a specific near-field strong interference source, using the heart dipole intensity $q_{\mathrm{ext}}$ as the variable. Compared with the case of no interference where $q_{\mathrm{ext}}=0$ nAm, represented by the dark blue line, the introduction of strong magnetocardiography interference where $q_{\mathrm{ext}}=10,000$ nAm, represented by the light blue line, similarly led to a decline in long-baseline performance. However, the physical mechanism differed significantly from that of environmental noise. The joint measurement array acted as a spatial high-pass filter in this context. To achieve a balance between preserving brain signals and canceling magnetocardiography interference with specific spatial correlations, the optimal baseline $d_{\mathrm{opt}}$ moved to a medium length range where $d_{\mathrm{opt}}\approx 5$ cm. The corresponding heatmap results in Figure 4D illustrates the global distribution of the SNR as a function of the baseline under different $q_{\mathrm{ext}}$ conditions. It was evident that as the external interference intensity increased, the high ${\mathrm{SNR}}_{\mathrm{array},\mathrm{linear}}$ region gradually shifted from long baselines toward the medium baseline range. The optimal baseline trajectory, indicated by the white dashed line, remained stably distributed at the scale of several centimeters rather than further contracting toward ultra-short baselines. This phenomenon indicated that for near-field point source interference with specific spatial correlation scales, an excessively short baseline would simultaneously weaken both the brain signals and the interference signals. A medium baseline could achieve a superior compromise between the spatial high-pass filtering effect and signal fidelity.

In summary, the simulation results indicated that the optimal baseline was not a fixed value but was dynamically adjusted according to changes in the dominant noise type. Under conditions dominated by environmental gradient noise, the optimal baseline tended toward an ultra-short scale of approximately 2 cm. Under conditions dominated by point-source physiological interference, the optimal baseline remained stably distributed within a medium scale range of approximately 2 to 5 cm.

### 3.2. Experiment 2: Validation of the Optimization Effectiveness of the BARO Algorithm in Complex Scenarios

**Figure 5:**
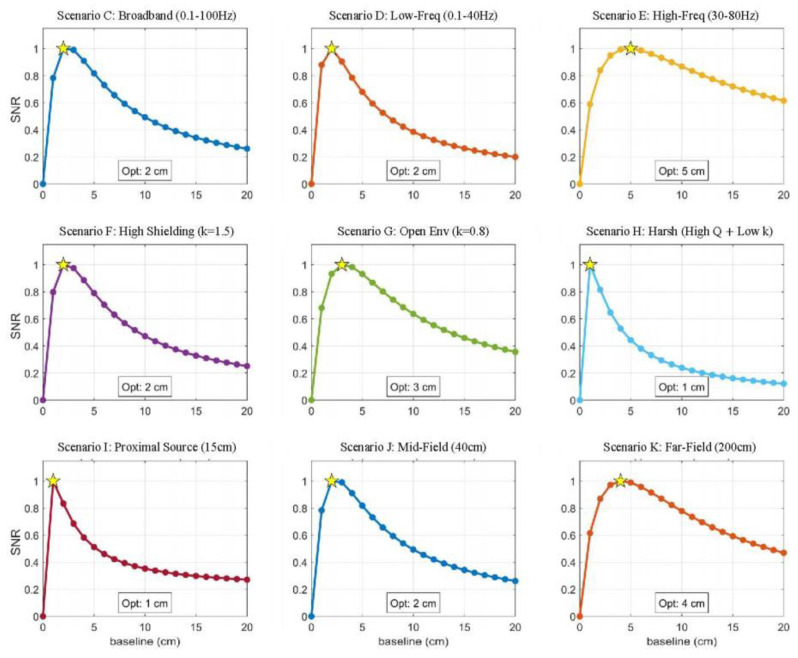
The optimal baseline optimization results in complex scenarios: (C–E) Optimal baselines for different task frequency band constraints. (F–H) Optimal baselines for different noise spectral slopes. (I–K) Optimal baselines for the spatial positions of different interference sources.

Figure 5 illustrates the optimal baseline selection results of the BARO algorithm under three categories of control variable conditions. The normalized signal to noise ratio curves exhibited distinct morphological differences and peak shifts, indicating that the optimal baseline $d_{\mathrm{opt}}$ possessed high sensitivity to task frequency band constraints in the first group, noise spatial structures in the second group, and interference geometric distributions in the third group. Specifically, the first group of experiments revealed the significant influence of frequency band constraints on baseline selection. For the standard broadband measurement in Scenario C with a range of 0.1 to 100 Hz, the optimal baseline determined by the algorithm was of medium length where $d_{\mathrm{opt}}=2$ cm. However, analysis targeting specific neural frequency bands showed significant differences. In the low-frequency ERF task of Scenario D with a range of 0.1 to 40 Hz, which was dominated by 1/f environmental noise, the optimization curve reached its peak prematurely at a short baseline where $d_{\mathrm{opt}}=2$ cm, reflecting the suppression requirement of the system for gradient noise amplification. Conversely, for the high-frequency Gamma band analysis in Scenario E with a range of 30 to 80 Hz, the SNR curve peak shifted to the right, producing a significantly extended optimal baseline where $d_{\mathrm{opt}}=5$ cm. This trend indicated that in the absence of strong low-frequency noise constraints, the system prioritized maximizing brain signal gain rather than gradient suppression.

The second group of experiments revealed the dependence of $d_{\mathrm{opt}}$ on the background noise spectral slope *k*. In the high-performance shielded environment of Scenario F where k=1.5, although the high-frequency attenuation performance was superior, the steep rise in low-frequency gradient noise limited the optimal baseline to a relatively short length where $d_{\mathrm{opt}}=2$ cm. It was noteworthy that this value was significantly shorter than the result of $d_{\mathrm{opt}}=3$ cm in the lightweight shielded environment of Scenario G where k=0.8, as the flatter noise spectrum of the latter permitted the use of longer baselines. In the adverse situation of Scenario H characterized by both high-intensity interference and poor shielding properties, the system entered a survival mode and compressed the optimal baseline to $d_{\mathrm{opt}}=1$ cm to maximize the common-mode rejection efficiency.

The third group of experiments elucidated the monotonic drift of the optimal configuration caused by the spatial position of external interference sources. Near-field interference in Scenario I at a distance of 15 cm caused the SNR to reach a sharp peak at a short baseline where $d_{\mathrm{opt}}=1$ cm, confirming the necessity of adopting a compact gradiometer architecture to counteract steep spatial gradients. As the interference source moved to the mid-field in Scenario J at a distance of 40 cm, the optimal baseline increased to $d_{\mathrm{opt}}=2$ cm. For far-field interference in Scenario K at a distance of 200 cm, the optimal baseline further extended to $d_{\mathrm{opt}}=4$ cm.

In summary, in all cases, the normalized signal-to-noise ratio presented a clear non-monotonic variation trend with baseline length and reached the maximum value within a finite baseline range. The significant differences in the optimal baseline positions under different scenarios reflected the comprehensive influence of task requirements, physical environments, and spatial distributions of interference sources on differential measurement performance. These results powerfully validated the capability of the BARO algorithm to adaptively select physically consistent optimal baseline configurations under multi-dimensional complex conditions, proving the universality and robustness of the method.

### 3.3. Experiment 3: Performance Evaluation of the Optimal Baseline in Source Localization Tasks

**Figure 6:**
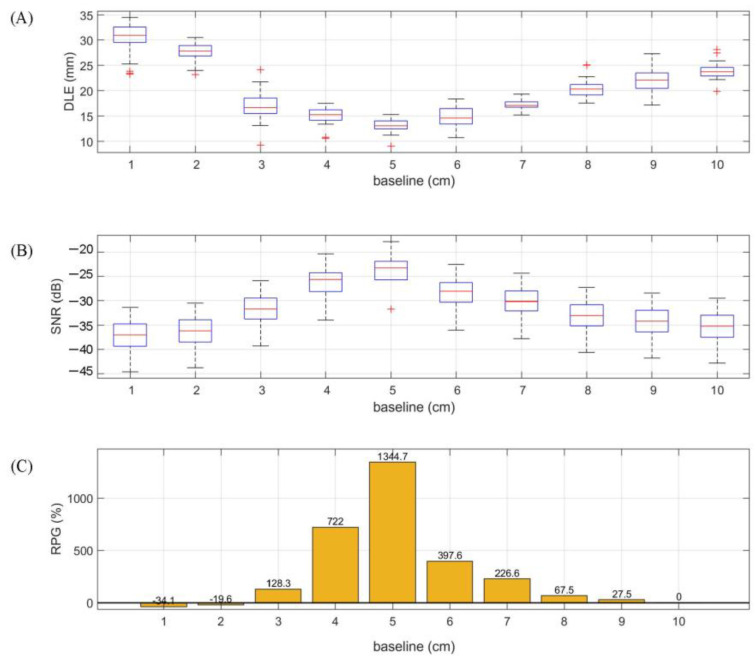
Performance evaluation results of source localization under Scenario E (high-frequency Gamma band, 30–80 Hz) for different baseline configurations: (**A**) The variation in DLE with baseline length. (**B**) The variation in SNR with baseline length. (**C**) The variation in RPG with baseline length.

Figure 6 illustrates the performance evaluation results of source localization under the conditions of Scenario E for different baseline configurations. This scenario corresponded to the high-frequency Gamma band analysis (30–80 Hz) in Experiment 2, where the optimal baseline was determined by the BARO algorithm as a relatively long baseline configuration with $d_{\mathrm{opt}}=5$ cm. This group of simulation experiments aimed to verify the manifestation of this task constraint mechanism in actual imaging from both the sensor level and the source level. Figure 6A provides the trend of the source-level indicator DLE as a function of baseline length, and it was observed that the optimal baseline obtained through BARO exhibited significant advantages in source localization accuracy. In the short baseline region where d<3 cm, the spatial resolution capability for the target source was limited because the differential measurement process provided insufficient effective gain for high-frequency weak magnetoencephalography signals, resulting in the DLE remaining at a high level. As the baseline length gradually extended, the DLE curve declined rapidly and reached its minimum value at d=5 cm, indicating that the spatial resolution capability of the array for the high-frequency Gamma source reached its optimum at this point, effectively suppressing systematic localization bias. When the baseline further extended outward, the DLE exhibited a gradually increasing trend, indicating that an excessively long baseline destroyed the gradient approximation condition for external interference sources. This allowed interference components that were not fully canceled to leak into the source space in the form of spatial artifacts, thereby causing a significant rebound in localization error. The ${\mathrm{SNR}}_{\mathrm{array},\mathrm{dB}}$ results provided in Figure 6B further reveal the underlying mechanism of the aforementioned localization accuracy improvement. It was observed that when the baseline length approached the optimal value of d=5 cm determined in Experiment 2, the array signal-to-noise ratio reached its maximum, and its value was significantly higher than those of the short baseline and excessively long baseline configurations. Compared with the short baseline region where d<3 cm, the array signal to noise ratio under the optimal baseline condition improved by more than 10 dB. This indicated that under conditions where the constraints of high-frequency Gamma bands and low-frequency gradient noise were relatively weakened, the system preferred to enhance the intracranial signal gain by extending the baseline length rather than prioritizing the enhancement of gradient suppression capability. This result was highly consistent with the phenomenon of the significant rightward shift of the peak in the normalized signal to noise ratio curve under Scenario E in Experiment 2. However, the presence of external interference gradients limited the infinite extension of the baseline length. d=5 cm constituted the optimal compromise point between brain signal capture capability and external interference suppression, which both avoided the insufficient signal gain brought by short baselines and prevented the introduction of significant interference artifacts by excessively long baselines. The RPG indicator shown in Figure 6C quantitatively characterizes the overall optimization effect of the BARO method in this scenario from the perspective of relative performance gain. The results indicated that compared with the reference fixed baseline configuration of d=10 cm, the optimal baseline achieved significant positive performance gains in Scenario E, directly reflecting the effectiveness of the baseline adaptive optimization strategy in high-frequency neural activity measurement and imaging tasks.

## 4. Discussion

In the OPM-MEG system, the introduction of reference sensors around the scalp array is a significant approach for enhancing noise suppression capabilities in complex electromagnetic environments while simultaneously preserving neural signals [29,30,31]. However, the spatial layout design of reference arrays has long lacked unified theoretical guidance; in particular, the setting of the synthetic baseline between the primary array and the reference array still relies primarily on prior experience. Departing from the traditional design philosophy of synthetic gradiometers that adopt fixed empirical baselines, this study proceeds from the statistical characteristics of environmental noise to systematically investigate the modulation mechanism of the synthetic baseline length on the measurement performance of the joint array.

The research results indicate that the synthetic baseline not only determines the sampling capability of the system regarding the spatial gradient of the environmental magnetic field but also plays a key role in balancing noise suppression efficiency and neural signal fidelity. On this basis, this study introduces a BARO-based baseline optimization strategy that enables the baseline length to be selected according to environmental noise characteristics. This strategy improves the overall detection capability for intracranial sources while effectively suppressing external interference and environmental gradient noise.

Figure 4 and Figure 5 systematically reveal the influence laws of synthetic baseline variations on array performance from the aspects of noise spatial structure, task frequency band constraints, and the spatial distribution of interference sources. The results demonstrate that the optimal baseline is not a constant but exhibits systematic variations according to environmental and task conditions. Furthermore, Figure 6 illustrates the performance of the BARO approach under multiple complex simulated scenarios. The results show that, compared with fixed empirical baseline configurations, the adaptive optimization strategy possesses significant advantages in both SNR enhancement and the improvement of source localization accuracy.

Notably, the BARO method is designed to provide a physically interpretable criterion for baseline selection in joint measurement arrays. While the simulations in this study adopt a simplified one-to-one symmetric layout to isolate the effect of baseline distance, the concept of an effective baseline may, in principle, be extended to more complex configurations. However, such extensions would require additional constraints and do not directly resolve the associated high-dimensional optimization problem. In configurations where the number or positions of reference sensors differ from those of the scalp array, the baseline can be interpreted as an effective distance parameter modulated by geometric factors. In this context, the BARO metric can be used to evaluate and compare different array configurations, providing guidance for interference suppression performance under given conditions.

Additionally, our findings have direct implications for the design of hardware integrated gradiometers. In current OPM development, integrating two sensing coils within a single compact housing is an emerging trend to reduce system complexity, particularly for short or ultra-short baseline gradiometers [32,33]. A critical design challenge for such sensors is determining the fixed physical distance between the internal sensing elements [34]. The BARO-based analysis provides a useful reference for this design choice by relating baseline length to environmental noise characteristics. By selecting an appropriate internal spacing at the design stage, the intrinsic noise-rejection capability of the hardware may be improved.

In summary, proceeding from the statistical characteristics of environmental noise, this study reveals the intrinsic modulation mechanism of the synthetic baseline on the performance of joint measurement arrays and proposes an optimization strategy based on the BARO method. The results highlight that the optimal baseline is not a fixed parameter but depends on environmental and task-specific conditions. While the present work does not fully resolve the broader problem of array design under realistic constraints, it provides a step toward incorporating environment-dependent factors into OPM-MEG sensor array configuration.

## 5. Limitations

The performance of the BARO method in this study was systematically evaluated primarily through numerical simulations. Although various noise spatial structures and interference geometric distributions were considered in the simulations, the non-stationary noise and more complex magnetic field gradient distributions prevalent in actual electromagnetic environments may pose higher challenges to the model assumptions. Another important limitation lies in the assumption of ideal sensor calibration. In practical OPM-MEG systems, calibration errors, sensor nonlinearity, and manufacturing tolerances may significantly affect noise suppression performance. In particular, shorter baselines tend to amplify sensitivity to calibration errors, which may shift the optimal baseline toward larger values in real-world applications. Incorporating calibration uncertainty into the modeling and optimization process will be an important direction for future work, in order to improve the robustness and practical applicability of the proposed approach. In addition, this study focuses on baseline optimization under fixed sensor configurations. Extending the BARO metric to full high-dimensional optimization problems, including sensor positions and orientations, remains challenging due to the non-convex and highly complex nature of the optimization space. Future work will explore the incorporation of additional constraints and regularization strategies to enable more tractable optimization in such settings, while preserving the physical interpretability of the baseline-related criterion.

## 6. Conclusions

This study addresses the issue that the determination of baseline length in OPM-MEG joint measurement arrays has traditionally relied on fixed empirical values by introducing an environment-driven baseline selection strategy based on noise and interference suppression (BARO). The proposed approach is used to identify suitable baseline configurations of synthetic gradiometers under different task and noise conditions, and its performance is systematically evaluated through numerical simulations. The results indicate that the optimal baseline is not a fixed value but varies systematically with task frequency band constraints, noise spatial structures, and interference geometric distributions. Within the simulated scenarios, the BARO approach suggests an effective baseline range of approximately 1–5 cm. Compared with conventional fixed-baseline configurations, the proposed strategy reduces the DLE by 15–18 mm and improves the ${\mathrm{SNR}}_{\mathrm{array},\mathrm{dB}}$ by 14–25 dB. These findings demonstrate that the BARO-based strategy enables environment-dependent baseline selection across a range of simulated scenarios and contributes to improved source imaging performance and robustness in OPM-MEG systems. While the present study focuses on baseline selection under simplified conditions, it provides a useful reference for incorporating environmental characteristics into the design considerations of OPM-MEG sensor arrays operating in complex electromagnetic environments.

## Data Availability

The data, aside from the data published in this manuscript, are not publicly available due to privacy restrictions.
